# Types of implementation of the dementia-specific case conference concept WELCOME-IdA in nursing homes: a qualitative process evaluation of the FallDem effectiveness trial

**DOI:** 10.1186/s43058-021-00191-0

**Published:** 2021-08-18

**Authors:** Sonja Teupen, Daniela Holle, Martina Roes

**Affiliations:** 1 German Center for Neurodegenerative Diseases (DZNE), Witten, Stockumer Straße 12, 58453 Witten, Germany; 2 School of Nursing Science, Faculty of Health, Witten/Herdecke University, Alfred-Herrhausen-Straße 50, 58455 Witten, Germany; 3 Department of Nursing Science, University of Applied Sciences (HS Gesundheit), Gesundheitscampus 6-8, 44801 Bochum, Germany

**Keywords:** Dementia, BPSD, Case conferences, Implementation, Complex interventions, Nursing Homes, Process evaluation, Qualitative research, Focus groups, CFIR

## Abstract

**Background:**

Dementia is regularly associated with behavioral and psychological symptoms of dementia (BPSD, also referred to as challenging behavior). Structured dementia-specific case conferences (DSCCs) enable nursing staff in nursing homes (NHs) to analyze and handle the BPSD of residents with dementia. The FallDem trial estimated the effectiveness of the structured DSCC intervention WELCOME-IdA (Wittener model of case conferences for people with dementia – the Innovative dementia-oriented Assessment tool) in NHs in Germany. No significant change in the overall prevalence of challenging behavior was found. A multipart process evaluation was conducted to explain this result.

**Methods:**

This qualitative process evaluation of the response of individuals, perceived maintenance, effectiveness, and unintended consequences was part of the multipart process evaluation that followed the framework by Grant et al. (Trials 14: 15, 2013). It used the data from semi-structured telephone interviews and focus group interviews with nurses and managers as secondary data. Selected domains of the Consolidated Framework for Implementation Research (CFIR) were used as deductive categories for a directed content analysis.

**Results:**

The interviewees in all NHs appraised WELCOME-IdA as generating positive change, although it proved important that some adjustments were made to the intervention and the organization. Thirteen CFIR constructs out of the domains *intervention characteristics*, *inner setting*, and *process* proved to be essential for understanding the different course that the implementation of WELCOME-IdA took in each of the four NHs. This is reflected in three types of WELCOME-IdA implementation: (1) priority on adjusting the intervention to fit the organization, (2) priority on adjusting the organization to fit the intervention, and (3) no setting of priorities in adjusting either the organization or the intervention.

**Conclusion:**

The unsatisfying results of the FallDem effectiveness trial can in part be explained with regard to the interplay between the intervention and the implementation which was revealed in the processes that occurred in the organizations during the implementation of the WELCOME-IdA intervention. Future implementation of WELCOME-IdA should be tailored based on an analysis of the organization’s readiness, resources, and capacities and should also define custom-made intervention and implementation outcomes to measure success. Furthermore, our results confirm that the CFIR can be used beneficially to conduct process evaluations.

**Supplementary Information:**

The online version contains supplementary material available at 10.1186/s43058-021-00191-0.

Contributions to the literature

- To date, the explanatory power inherent in the interplay of an intervention and its implementation has received little empirical examination in process evaluations.
- We use the analysis of this interplay to explain the unsatisfying results of an effectiveness trial by identifying which factors became relevant during the implementation of a complex intervention and under which circumstances.
- We demonstrate the benefits of the Consolidated Framework for Implementation Research (CFIR) for an analysis of the interplay between an intervention and its implementation by focusing on cross-connections between the CFIR’s single domains, thus confirming its applicability in process evaluations under complex conditions.

## Background

Dementia is regularly associated with behavioral and psychological symptoms of dementia (BPSD) [1]. Among persons with dementia living in nursing homes (NHs), the highest prevalence is found for agitation and apathy [2]. BPSD are also referred to as challenging behaviors due to their potentially negative impact on the person with dementia, other residents, relatives, and nursing staff [3–5]. For the management of BPSD, psychosocial interventions are given priority over pharmacological treatment [6]; such interventions include, e.g., personalized or group activities, manipulation of environmental cues, staff training, and case conferences [7–9]. Staff training and case conferences are considered system-level interventions that enable sustainable change [8].

### The FallDem trial

**Fig. 1:**
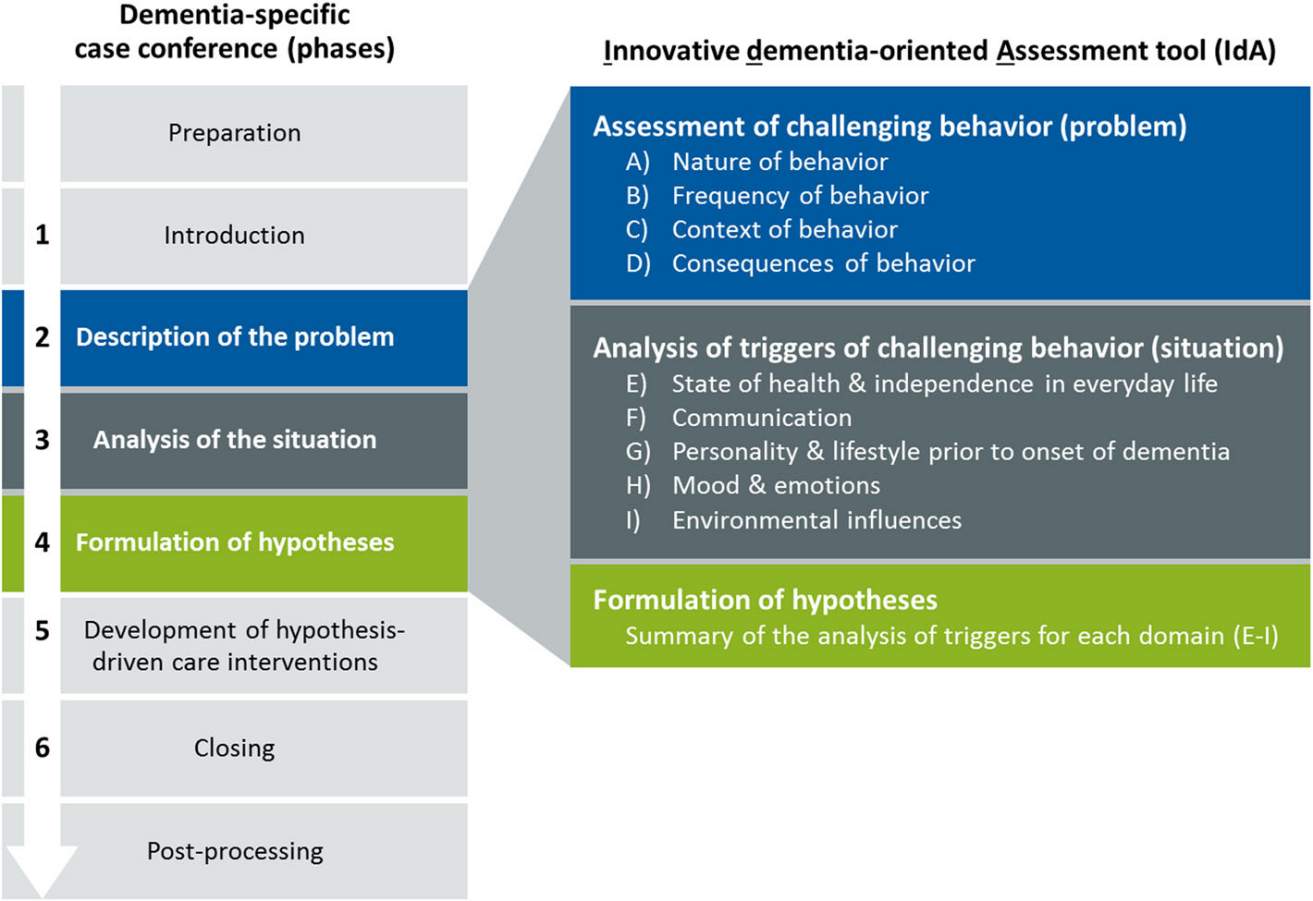
Process structure of DSCC in WELCOME-IdA and domains of the IdA

The FallDem trial studied the effectiveness of WELCOME-IdA (Wittener model of case conferences for people with dementia – the Innovative dementia-oriented Assessment tool) [10] in NHs in Germany [11–13]. The systematic, goal-oriented dementia-specific case conference (DSCC) intervention WELCOME-IdA is theoretically based on the Need-Driven Dementia-Compromised Model [14] and on concepts of collective learning in organizations [15]. It was developed based on a literature review [9], expert consultations [15], and a feasibility study [16]. The DSCC in WELCOME-IdA enables nursing staff in NHs to *analyze* factors that influence BPSD and to *develop* individualized care interventions. Central within each DSCC (Fig. 1) is behavior analysis using the Innovative dementia-oriented Assessment tool (IdA) (details on the WELCOME-IdA intervention are presented in [17]).

The IdA supports phases 2–4 of the case conference by providing 9 domains for the in-depth assessment of behavior in its context.

The FallDem trial used a stepped-wedged cluster randomized controlled trial (cRCT) design [18]. The results were in part unsatisfying: No significant change in the overall prevalence of challenging behavior (primary outcome) was found. For staff, the mean score for work-related burnout (secondary outcome) significantly decreased from the control phase to the intervention phase (−4 points; 95% confidence interval (CI) −7.3 to −0.3; *p*=0.032). No change was observed concerning the work-related stress of staff (secondary outcome) [13].

As a hybrid type I study, the FallDem trial included an implementation strategy [11]. In addition to the three consecutive components of the WELCOME-IdA intervention (in-service training in performing DSCCs, DSCCs with support (on-the-job training), and DSCCs without support [10, 17]), the implementation strategy consisted of information and kick-off meetings for the NH management and nursing staff; in-service training on the topics of dementia, challenging behavior, and moderator skills; the formation of a steering group consisting of managers and nursing staff that was responsible for the implementation process; telephone reminders to support the preparation of each DSCC; and a support hotline (details on the implementation strategy are provided in [10]).

### Process evaluation of the FallDem trial

Delivering and implementing a complex intervention in a NH is challenging, so it is necessary to interpret the observed degree of effectiveness against the background of potential implementation errors. Factors may lie in the design of the research study, in the intervention itself, and in the implementation processes [19, 20]. Thus, a multipart process evaluation was conducted in parallel to the effectiveness trial [10] following Grant et al.’s [21] framework. This evaluation comprised three perspectives: (1) Analysis of the *context*, *reach*, and *recruitment of participants* and *delivery of intervention* showed inaccuracies in the implementation of WELCOME-IdA and methodological limitations of the effectiveness trial [17] (Table 1). (2) Analysis of the *response of the NHs* (clusters) provided insight into which elements of the intervention were adopted and which were adapted, concluding that WELCOME-IdA needs to include more possibilities for the tailored adaptation of the intervention [22]. (3) This article analyzes the *response of individuals* (staff) towards the implementation of WELCOME-IdA and *perceived maintenance* processes within the NHs during the implementation phase. It aims to shed light on the perceived individual implementation processes in the different NHs in order to come to conclusions regarding the *effectiveness* on the trial outcomes and the *unintended consequences.* It reports the analysis of *factors influencing the implementation*. Our research question was: *What was the response of the individuals (staff) towards the implementation of WELCOME-IdA and how did this influence the implementation of WELCOME-IdA in NHs?* In combination with the previously generated results, this article also aims to answer the question: *What influence did the implementation have on the trial results?*

**Table 1:** Characteristics of participating nursing homes and selected results of the first part of the process evaluation of the FallDem trial [14]

Nursing home	E29	E79	E75	E82
Number of residents at baseline (*n*)	79	100	80	54
Number of units at baseline (*n*)	3	4	2	2
Case conferences prior to WELCOME-IdA implementation	Yes Weekly 1–2 h	Yes Quarterly 1 h	Yes Quarterly 1 h	No
Number of participants per DSCC (recommended in WELCOME-IdA: 5–8) (*n*)	8–19	7–17	6–9	4–6
No prior case conferences or less often, thus difficulty of integrating WELCOME-IdA DSCCs into existing routines.				
High levels of sick leave and high workload and time pressure, thus difficulty of integrating WELCOME-IdA DSCCs in routine care.				
No continuous participation of the same staff members in the consecutive parts of WELCOME-IdA, thus difficulty of establishing learning processes and radiation effects, possible delay of change in nursing staff behavior, and prevalence of BPSD of residents.				
Low number of target residents reached in the intervention (residents were discussed twice, nursing staff selected residents who were not included in the study sample, reduced number of DSCCs, dropout of two clusters); thus, the required power >80% was not reached.				
Reduced frequency of WELCOME-IdA DSCCs, what might have had an impact on the effectiveness of WELCOME-IdA.				
Nursing staff selected residents for DSCC with relatively low score of behavioral disturbances, what might have had an impact on the effectiveness of WELCOME-IdA.				
In some cases, no care interventions addressing BPSD were planned during the DSCC, what might have had an impact on the effectiveness of WELCOME-IdA.				

## Methods

This study applied a qualitative research design using qualitative process evaluation data as secondary data and focusing on implementation knowledge, particularly barriers and facilitators [23, 24]. The Consolidated Criteria for Reporting Qualitative Research (COREQ) [25] are applied (see Supplementary Table 1).

### Study sample and data

Thirty-four longitudinal semi-structured telephone interviews with 9 head nurses and 15 retrospective focus group interviews with a total of 146 participants from different participant groups (DSCC participants from nursing teams, DSCC moderators, and steering groups) were conducted in 4 NHs in parallel to the effectiveness trial between 2013 and 2015 (characteristics of interviewees are reported in [22]).

The aim of the telephone interviews (duration: 15 min on average; min 7, max 24) was to enquire about the preparation and post-processing of the DSCCs, alterations made to the intervention, experiences with the intervention, and changes in residents’ behavior and nursing staff response. At the end of the intervention phase, focus group interviews (duration: 40 min on average; min 25, max 61) were conducted to learn about participants’ experiences with the intervention and the implementation strategy [22].

### Analytical framework

Process evaluations can be used to explain trial results and to improve implementation strategies [24, 26]. The particular strengths of a qualitative process evaluation are that it contributes to understanding social processes that occurred during implementation [27], helps explain when observed outcomes are divergent from expected outcomes, and sheds light on contextual factors that influenced the implementation and may have led to variations in effectiveness [28]. The Grant et al. framework was supplemented by the Consolidated Framework for Implementation Research (CFIR) [29] as analytical framework, which has been used in implementation research and process evaluations [30–33], to introduce a clear focus on factors that influenced the implementation process. The CFIR represents a consolidation of major implementation theories, providing consistent definitions of concepts. It comprises 26 constructs (plus 15 sub-constructs) constituting 5 domains of determinants [34]. The domains *intervention characteristics*, *inner setting*, and *process* were selected for the analysis (see Supplementary Table 2 for associated constructs). The domains *outer setting* and *characteristics of individuals* were dropped because the data did not include detailed information regarding these domains.

### Analysis

The three selected CFIR domains and the associated constructs were used as deductive categories for a directed qualitative content analysis [35]. The results were interpreted in light of the underlying theoretical concepts. Topic-oriented and case-oriented approaches were applied, leading to the identification of implementation types [36].

**Fig. 2:**
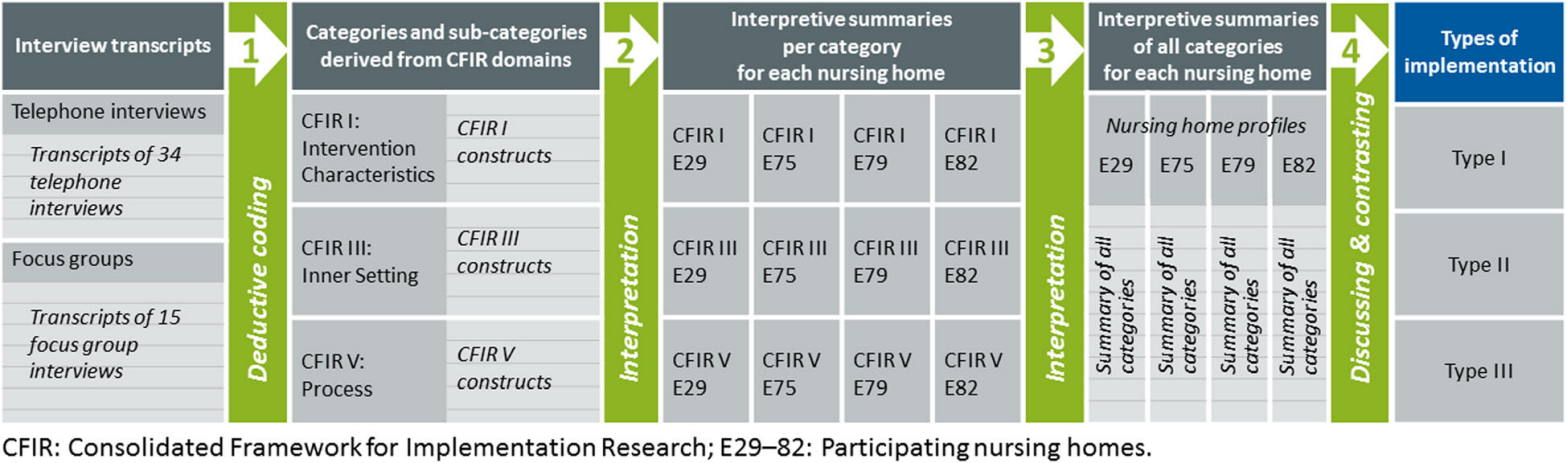
Process of analysis, interpretation, and identification of implementation types

All interviews were audio-recorded, transcribed verbatim, and organized using MAXQDA 2018 software. The interviews were coded by ST. ST, DH, and MR discussed the codings. The level of analysis was the individual organization (four NHs). For each NH, summaries per category and sub-category (CFIR constructs) were written and interpretive case summaries were developed. ST, DH, and MR interpreted the results and drew comparisons between the different groups involved in implementation within and between the individual organizations. Finally, implementation types were built (Fig. 2).

## Results

Thirteen CFIR constructs proved significant for understanding the different course that the implementation of WELCOME-IdA took in each of the four NHs and for distinguishing the types of WELCOME-IdA implementation (Table 2).

### Response of individuals, perceived maintenance, and influencing factors

#### CFIR domain I: intervention characteristics

##### Relative advantage

WELCOME-IdA as such was considered helpful and effective (E29, E75, E79), at least in the beginning and in relation to smaller aspects of residents’ behaviors (E82). In E29, E79, and E75, a range of positive effects was perceived on four levels: the individual staff member, the team, the resident, and the organization, e.g., in three NHs (E29, E79, E75) change of nursing staff’s view of the residents and increased understanding and appreciation of the residents’ needs and behaviors was reported: “You might see the behavior […] in a completely different way [...] we had a resident who was extremely difficult to care for [...] to see that this aggression that she has is also to be seen as a strength [...] because she can't do anything anymore, in terms of taking care of herself or participating in activities, but the aggression and this defending herself, that is still something” (E29_ZI_M, 127-131). E75 interviewees reported an improved ability to bear situations and behaviors that could not be changed: “What I also find very successful is when colleagues, it sounds stupid, but when colleagues somehow realize ‘Okay, there's nothing I can do about it now. I have to be able to bear it” (E75_ZI_M, 111). E82 interviewees did not report similar positive changes in team cohesion and communication between teams and no positive change in communication with management and did not perceive any reduction in their stress but rather mentioned the additional burden of individualized interventions: “In fact, it’s more work, but it’s there now; the main thing is that the resident feels comfortable” (E82_ZI_WB1+2, 262).

##### Adaptability

For the head of the NH E29, adopting WELCOME-IdA was not the main focus. Rather, participating in the trial was seen as a test run and as a blueprint for a case conference model to be developed later in light of the experience gained. The number of IdA domains to be processed in a single DSCC was reduced [22]. In E79 and E75, several adjustments were made, e.g., role-taking by leaders instead of nursing staff (E79, E75) and building pairs of nurses for preparing a case with the IdA assessment sheets (E79). The DSCCs in E82 were mostly conducted as intended, e.g., all IdA domains were completed during the DSCCs. One major deviation was that relatives of residents were included.

##### Complexity

NHs (E29, E79, E75) perceived WELCOME-IdA as highly complex and difficult to implement due to the extensive new assessment instrument and the hermeneutic perspective that had been unfamiliar to most of the nursing staff. Thus, a lengthy period of practicing and learning by doing was deemed necessary before the DSCC could be performed as intended. In E29, the IdA was perceived as too extensive with regard to the number of IdA domains to be processed in a single DSCC. In E79 and E75, some components of the intervention were perceived as too difficult to apply for the intended target group: the different roles during the DSCC (E79, E75), assessing the situation of a resident while preparing a DSCC (E79), participating in a DSCC without slowly getting acquainted with the intervention (E75), and overcoming possible fears in advance (E75). In E82, the extensiveness of the IdA was considered key: “this is how you really take a close look at the resident” (E82_ZI_WB1+2, 68).

#### CFIR domain III: inner setting

##### Tension for change

The heads of the NHs E29 and E82 saw the FallDem project as a free-of-charge personnel development opportunity. The heads of E75 and E82 mentioned a need for an adequate case conference model for residents with dementia (E75) and challenging behaviors (E82). The head of E79 articulated that the motivation for participating in the trial was to be part of research innovations, to stand out from other providers and to generate staff commitment.

##### Relative priority

A mixed priority was given in E29, where the head of the NH reported that it was first necessary to enforce the project against the managerial colleagues’ views. In other NHs, relative priority could be seen in statements regarding the steering group, an important element of the implementation strategy: In two cases, in addition to members intended by WELCOME-IdA, the steering group comprised representatives of quality management (E79, E82) or the provider (E79). In E82, at the same time, a rather low priority was reflected in the kind of leadership engagement (see below). In E75, an ambivalent priority of the implementation was reflected in that the steering group readily accepted it from the beginning but then decided to not meet again after the completion of the facilitated steering group meetings.

##### Learning climate

In E29, new or inexperienced staff were given separate additional internal training instead of being integrated into the running DSCCs to learn by doing. In E79, inexperienced staff were integrated into the running DSCCs and the managers successively stepped back from organizing the process. Statements of the E75 steering group reflected relatively low confidence in the nursing staff’s competences. In E82, nursing staff interviewees acknowledged that contrary to what might have been expected, all DSCC participants contributed actively.

##### Leadership engagement

Leadership engagement was reported with regard to different steps in the implementation process. According to the nursing teams in E29, the initial commitment of the head of this NH was characterized by “little transparency, little engagement, and little guidance” (E29_ZI_WB1, 184). The steering group took on responsibility for the general coordination of the implementation, while responsibility for the actual implementation was delegated to the “interface coordinator” (see below). The engagement of the head of this NH increased only later in response to external motivation by the interface coordinator, which is contrary to the head’s own view of being the motor. In E75 and E79, all leaders committed to the project in the beginning and in E75 all roles within the intervention were taken on by leaders. From the E82 nursing staff’s point of view, the head of this NH did not show much engagement in the implementation. Additionally, the statements of the head of the NH can be perceived as slightly disparaging in relation to WELCOME-IdA: the assessment sheets had been “annoying” “only at first sight” (E82_ZI_SG, 119), and the strict structure of the DSCCs had seemed “sometimes a bit […] ridiculous” (E82_ZI_SG, 184).

#### CFIR domain V: process

##### Opinion leaders

Besides the engagement of the heads of the NHs, the heads of nursing wards in E29 and E79 endorsed the implementation, while the head of the NH E75 was critical regarding the perceived low quality of the implementation plan and the staff training. In E82, senior colleagues manifested themselves as opinion leaders in that they displayed some resistance to the new care interventions: “The older ones say ‘Oh, it's no use anyway,’ and so on” (E82_ZI_WB1+2, 325).

##### Formally appointed internal implementation leaders

In E29, a person who was trained as moderator for the DSCC was appointed as an “interface coordinator” who led the actual implementation and carried out additional internal training and in E79, the heads of the nursing wards were appointed the role of “linkages” (E79_ZI_SG, 111) between nursing teams and the steering group. In E75 and E82, all responsibility for and control of the implementation was maintained at the level of the steering group.

##### Innovation participants

In WELCOME-IdA, innovation participants are not the residents but the staff members who participate in the DSCC. They were engaged quite differently in terms of training and information. In E29, staff members were trained as intended and were obliged to participate. E79 and E82 tried to train as many staff members as possible, a deviation from WELCOME-IdA. In E75 and E82, the nursing staff members did not feel sufficiently informed about the project and their individual participation in it: “We as ordinary staff got it all served” (E75_ZI_WB2, 170). At the end of the implementation phase, E82 nursing staff articulated insecurity about whether and how their organization planned to continue with WELCOME-IdA.

##### Executing

E29 and E79 continued conducting DSCCs until the end of the implementation phase [17], they however showed adaptations in the execution in that they independently spread the implementation to a third ward and the adult day care (E29) or the remaining nursing wards to “leave nobody out in the cold” (E79_ZI_SG, 58) (E79). The E79 steering group strongly emphasized receiving feedback from the project team on the organization’s performance and on achieving the trial’s outcomes to make sure they were “going in the right direction” (E79_ZI_SG, 105). E75 did not continue conducting DSCCs as planned until the end of the implementation phase but reduced the frequency by half [17]. The steering group in this NH decided not to meet again after the completion of the facilitated steering group meetings. In E82, a major deviation was that relatives of residents were included in the DSCC. E82 did not continue conducting DSCCs after the completion of the on-the-job trainings [17].

### Types of implementation

Underlying the different course of implementation in the four NHs was an interplay between the intervention and the implementation that triggered processes that were implied by neither the intervention nor by the implementation strategy alone. The findings suggest that the relation between organization and intervention can help explain the results of the trial. Three types of WELCOME-IdA implementation were identified that represent the three different ways of configuring this relation. The central distinguishing feature is how the NHs gave priority to adjusting either the intervention or the organization to establish a fit between them. That includes the process result (*how*) and the factors that contributed to the described development (*which*). The types of WELCOME-IdA implementation are as follows:

- Type I: Priority on adjusting the intervention to fit the organization;
- Type II: Priority on adjusting the organization to fit the intervention;
- Type III: No setting of priorities in adjusting either the organization or the intervention.

#### Type I: Priority on adjusting the intervention to fit the organization

This type of WELCOME-IdA implementation is represented by E29. One could say that this NH took the fast track. The head of the NH viewed certain aspects of the intervention as highly relevant for the organization. The (hidden) aim of E29 was to develop an organization-specific way of conducting DSCCs and to spread it throughout the organization as soon as possible. Less emphasis was placed on the sustainability of WELCOME-IdA itself and on achieving the trial’s outcomes. Consequently, E29 primarily adjusted the intervention to the prerequisites (e.g., forms of communication), needs (e.g., staff training), and goals (e.g., own DSCC concept) of the organization. The intervention was made less complex (e.g., reducing IdA domains), and the organization departed from the implementation plan of the trial to achieve a rapid spread across all wards.

#### Type II: Priority on adjusting the organization to fit the intervention

The second type of WELCOME-IdA implementation is represented by E79 and E75. Both NHs were eager to do it right. Any changes were made with the aim of implementing the intervention precisely. E79 strongly emphasized adjusting the organization to make it fit the needs of the intervention (e.g., expanding the steering group, building pairs for preparation) because the intervention was seen as highly relevant in exactly the form that had been suggested. Adjustments to the intervention were made only to reinforce its original purpose (e.g., expanding staff training, role-taking by leaders). E75, too, emphasized adjusting the organization (e.g., parallel structure of working on the issue of challenging behavior in the steering group meetings and the regular team meetings, reducing the frequency of DSCCs) but was also willing to adjust parts of the intervention if necessary (e.g., role-taking by leaders instead of nursing staff). In both NHs, the leaders, such as the head of the NH and the heads of the nursing wards, showed a high degree of engagement. Responsibility was initially kept at the managerial level and only successively (E79) and hesitantly (E75) delegated to the nursing staff on the ward level. Great effort was exerted at the organizational level to implement the intervention as planned; workflows were restructured around the requirements of the intervention in both NHs.

#### Type III: No setting of priorities in adjusting either the organization or the intervention

This third type of WELCOME-IdA implementation is represented by E82. This NH tried both routes but only to a certain extent and without prioritizing one of the two ways, that is, (1) adjusting the intervention (e.g., inviting relatives to the DSCC; using the DSCC format for something other than intended; securing communication of DSCC results but waiving systematic evaluation) and (2) adjusting the organization (e.g., reinforcing the importance of the intervention but showing little leadership commitment; training many staff but neglecting information flow thereafter). There was no strong communication or participation culture established that could have enabled the commitment and development of staff. Instead, staff members were not sufficiently informed and were confronted with adverse attitudes towards the intervention at different levels of their organization. One answer to the question why the head of the NH lost interest during the implementation process could be that E82 was the only NH that clearly mentioned the reduction of challenging behavior as one objective and motivation for participating in the trial. A previous attempt to implement case conferences in this NH had failed because there had been too little time, staff had not been sufficiently informed, and overly difficult cases had been selected. It is possible that this motivation decreased when it became clear that this outcome would, again, not be achieved to a satisfactory extent and within a reasonable time. In the end, no fit between the organization and the intervention and ultimately no sustainability could be achieved.

## Discussion

### Effectiveness on trial results and unintended consequences

The characteristics of an intervention, as described in the CFIR domain *intervention characteristics*, influence the course and success of implementation efforts [29, 37]. In addition to this known unilinear relation, our process evaluation revealed an *interplay between the intervention and the implementation*: (a) the subjective perception of the intervention influenced the implementation process in each NH, and (b) the implementation process created conditions that became influencing factors for the components of the intervention.

With regard to the subjective perception of the intervention, two factors proved particularly influential: the perceived complexity and the perceived impact of the intervention. It was important, first, that the underlying instrument IdA was not perceived as too complex and, second, that the DSCCs led to positive effects in daily practice. The analysis showed that the estimation of the intervention was similar—and in many aspects positive—across participant groups and across NHs. While complexity was in part perceived as too high and the intervention was adapted accordingly, mainly positive effects in terms of change were perceived. All NHs in our sample reported considerable positive effects, even if they were not or only partially visible in the statistical analysis [13]. This confirms the results of other process evaluations of similar complex interventions for dealing with BPSD in NHs. In the process evaluation of the *Grip on Challenging Behavior* care program for managing challenging behavior in NHs which also included a systematic analysis of behavior in its context, Zwijsen et al. identified the perceived overwhelming extent of the care program as a barrier to implementation [23]. Conversely, Nakrem et al. [38] found the perceived feasibility of the innovation to be a facilitating factor for the implementation of a team-based approach to care, including geriatric assessments and structured case conference meetings. Additionally, in the process evaluation of the multicomponent intervention *Targeted Intervention Interdisciplinary Model for Evaluation and Treatment of Neuropsychiatric Symptoms* (TIME), which included case conferences to reduce agitation in persons with dementia in NHs, Lichtwarck et al. [39] found that the feature of TIME that it was perceived easy to grasp and working effectively was decisive for implementation success.

In addition to intended consequences that were in line with the trial’s outcomes, our results showed unintended consequences [21] of WELCOME-IdA, which might have influenced the effectiveness study. The change in view of residents and the change in understanding and appreciation of residents could be seen as an intended result of WELCOME-IdA because the intervention aims at establishing a specific case-analytical and hermeneutical perspective and at initiating dynamics of collective learning. These were, however, not included in the trial’s outcome measures. This is related to the change in perception and evaluation of challenging behavior. It is possible that these consequences on the side of the individual staff members influenced the trial results because that what was measured in the trial was affected by the change in perspective, e.g., apathy gained meaning as a BPSD when the nursing staff’s focus was directed towards a deeper reflection of the resident’s needs. Also change in team cooperation and change in communication between teams and managers might have influenced the trial results because for the nursing staff, new possibilities of discussing problematic situations opened up, again, possibly leading to an increased focus on these situations and to a lower willingness to see BPSD as an individual problem and as unchangeable. Finally, the reported ambivalent change of challenging behaviors and the additional burden from the intervention can be interpreted as harmful unintended consequences that might have influenced effectiveness negatively. In summary, it has become clear that WELCOME-IdA is truly a systems-level intervention and that it is difficult to identify clear consequences of and relationships between the reported influencing factors.

The process evaluations referred to above revealed, as additional critical facilitating factors, a high degree of engagement of managers and champions, sufficient time resources, and sufficient staff training [23, 38, 39]. Although the three studies applied a different analytical framework than the CFIR, these highlighted facilitating factors can be related to the CFIR domains *inner setting* and *process*. These studies and our study show that the *assessment* of the intervention by NHs has to be interpreted in light of organizational (e.g., leadership engagement) and procedural constructs (e.g., engaging of innovation participants) to account for the systemic character of the DSCC intervention. Our results also show that *adjustments* made to the intervention must be understood in this light. In this respect, it is important to interpret organizational and procedural factors in relation to perspectives on the characteristics of WELCOME-IdA, such as its complexity. Finally, our study showed that organizational and procedural factors must be seen in connection with each other. Consequently, the differences found in the implementation processes in the four NHs cannot be approached by focusing only on the differences in perspectives on the characteristics of WELCOME-IdA.

What our process evaluation contributes to the literature is the following: Even if an intervention is perceived by the participants as positive and as having a positive impact, it is possible that the implementation process stagnates or is discontinued. On the other hand: even if the intended outcomes of an intervention are not achieved during the trial, the implementation process can still continue and be perceived as successful. By taking different CFIR domains into account, the complex interplay between the intervention, and the implementation within the context of the organization was identified as decisive. For example, during implementation, when it turned out that the trial’s outcomes would not be reached, some NHs developed a different understanding of changing challenging behavior and started to focus on alternative outcomes, such as perspective change in staff. However, only when matching between the intervention and the individual organization could be reached was it possible to continue implementation, albeit in a way that deviated from the study protocol. We already found in the second part of the multipart process evaluation that the intervention should have allowed more possibilities for adaptation to the individual NHs [22]. By looking deeper into the qualitative data using the CFIR as an analytical frame, we could understand that there are at least two ways of establishing a customized approach between the intervention and the organization and, accordingly, at least two ways of reaching a tailored implementation strategy that considers the interplay between intervention and implementation within the organizational context. One way is to adjust the intervention to fit the prerequisites and requirements of the organization. The second way is to adjust the organization to fit the prerequisites and requirements of the intervention. Our results show that for the implementation of WELCOME-IdA, it was important for the NHs—in the sense that something has emerged from the interplay of intervention and implementation that individuals perceived as positive—to prioritize one of these two strategies. Both paths allow organizations some flexibility, but which path is the most appropriate strongly depends on organizational characteristics. However, another way is possible in which intervention and implementation are equally adjusted. This possibility of a type IV (priority on adjusting intervention and organization alike) has not been shown in our data, but seems plausible. Offering some degree of flexibility to *adjust an innovation* to the needs and prerequisites of the implementation context is generally recommended for complex interventions [19], has proven important in process evaluations of similar interventions [38, 39], and has recently been confirmed by a systematic review of barriers and facilitators of complex interventions for persons with dementia in long-term care [40]. The possibility of *adjusting the organization* to fit the intervention has not yet been well explored in research, but this could be realized with the CFIR as an analytical framework.

### Implications for research

First, our results are relevant for effectiveness studies of complex interventions. The perspective on the interplay between intervention and implementation influences the definition of implementation outcomes in addition to intervention outcomes. Our results confirmed the need for a respective paradigm shift in psychosocial dementia care research [20]. Considering all findings from the process evaluation of the FallDem trial [17, 22], it becomes clear that the FallDem cRCT had to deal with aspects of an implementation error, although it included a general implementation strategy that was supposed to be concretized by the participating NHs. Our results indicate that it is essential to integrate implementation knowledge from the very beginning to assess potential facilitators and barriers within the individual context early on (e.g., feasibility study). The results of such an assessment could be considered in planning an effectiveness trial. In addition, if an implementation perspective throughout the study is to support the adjustment of the intervention if necessary, this must be considered in the effectiveness design (e.g., SMART design [41], hybrid design [42]). In light of our findings, a hybrid type III effectiveness-implementation study [41] is a promising next step for promoting the WELCOME-IdA intervention. On the one hand, such a study could focus on implementation outcomes such as those suggested by Proctor et al. [43]: acceptability, adoption, appropriateness, feasibility, fidelity, implementation cost, penetration, and sustainability. On the other hand, regarding effectiveness, it could concentrate on outcomes on the staff and team levels, such as changed perspective, appreciation of residents’ situation and behavior, communication, and team cohesion, as observed previously [39, 40]. As our results indicate, the CFIR can be used to define these implementation and effectiveness outcomes [30, 40].

Second, our results are relevant for implementation research. We applied the CFIR as an analytical framework by using its constructs as categories for deductive coding, as has been done before [30, 40]. This proved the CFIR’s potential to study the process (*how*) and influencing factors (*which*) simultaneously. While some studies select one CFIR domain on the basis of theoretical considerations [44–46], this study, based on a qualitative analysis, provided insights into the CFIR constructs most relevant for our study. Powell et al. [47] recommend systematically selecting and tailoring implementation strategies on empirical grounds. In this respect, recent implementation literature discusses the benefits of qualitative methods for tailoring an implementation [48–50]. We gradually detached our interpretations from the individual CFIR constructs and integrated the findings into comprehensive case accounts. The advantage was that key aspects were contextualized and cross-connections made visible. Nonetheless, it proved beneficial to finally return to the CFIR constructs in order to consolidate the interpretations in a structured way, to compare our results with those of other research and to derive practicable implications for future implementation.

### Limitations

The FallDem trial included a process evaluation based on Grant et al. [21]. We used the qualitative data for a secondary data analysis and applied the CFIR as additional analytical framework. Thus, coding was in part independent from the interview purpose and questions. Using the CFIR for both data collection and analysis has advantages over using it only for analysis [30]; however, detaching the analysis from the interviews’ rationale enabled further interpretations.

Due to the trial’s stepped-wedge design, there were no follow-up data available regarding the maintenance of and experiences with WELCOME-IdA after the intervention phase [22].

The process evaluation concentrated on neither the content of the DSCCs (forms of challenging behavior, discussion processes, types of care interventions, etc.) nor the realization of the planned care interventions; thus, only limited conclusions can be drawn regarding how this possibly influenced the implementation [17]. We assume that the interviewees would have addressed this if it had been more relevant to them, but future implementation of WELCOME-IdA should consider these aspects.

## Conclusion

The process evaluation of the FallDem trial revealed a critical interplay between the WELCOME-IdA intervention and its implementation. It reconstructed key elements of this relationship and showed how these elements became relevant, influenced each other, and exerted potential systemic influence on the trial results. The analysis could only approximate this influence through the traces that became apparent in the reported consequences. However, by comparing different types of WELCOME-IdA implementation, it identified two beneficial ways of successfully implementing WELCOME-IdA in NHs: prioritizing the adjustment of either the intervention or the organization. Which path an NH chooses depends on the existing structures and characteristics of the organization. Thus, any implementation of WELCOME-IdA should start with an analysis of the organization that considers organizational aspects described in the CFIR. The discussion of this analysis with NHs should address the reasons for consideration and the challenges that the characteristics yield in order to reach an informed decision on which way of implementing is the most suitable.

## Supplementary Information

**Additional file 1.**

**Additional file 2.**

## Data Availability

The datasets generated and analyzed during the current study are not publicly available due to legal regulations of the German Center for Neurodegenerative Diseases.

## Abbreviations

BPSD: Behavioral and psychological symptoms of dementia
CFIR: Consolidated Framework for Implementation Research
COREQ: Consolidated Criteria for Reporting Qualitative Research
cRCT: Cluster randomized controlled trial
DSCC: Dementia-specific case conference
IdA: Innovative dementia-oriented assessment tool
NH: Nursing home
WELCOME-IdA: Wittener model of case conferences for people with dementia – the Innovative dementia-oriented Assessment tool
