# Supplementary material for: Types of implementation of the dementia-specific case conference concept WELCOME-IdA in nursing homes: a qualitative process evaluation of the FallDem effectiveness trial
Source: Implement Sci Commun. 2021 Aug 18;2:90. doi: 10.1186/s43058-021-00191-0 (PMC8371834; doi:10.1186/s43058-021-00191-0)
Supplement: Supplementary file 1 — Additional file 1. [file 43058_2021_191_MOESM1_ESM.docx]

**Supplementary Table 1. Consolidated criteria for reporting qualitative studies (COREQ): 32-item checklist.**

| **Topic and Item No.** | **Guide Questions/Description** | **Comment** |
| --- | --- | --- |
| **Domain 1: Research team and reflexivity** | | |
| Personal Characteristics | | |
| 1. Interviewer/facilitator | Which author/s conducted the interview or focus group? | Telephone interviews: DH et al.  Focus group interviews: MR et al. |
| 2. Credentials | What were the researcher’s credentials? | ST, PhD, MA Social Sciences  DH, PhD, MScN, RN  MR, PhD, MA Sociology, RN. |
| 3. Occupation | What was their occupation at the time of the study? | ST: Research Associate  DH: Research Associate  MR: Full Professor, site leader, group leader. |
| 4. Gender | Was the researcher male or female? | Female. |
| 5. Experience and training | What experience or training did the researcher have? | The researchers had extensive experience in face-to-face interviews. They had also supervised qualitative research. |
| Relationship with participants | | |
| 6. Relationship established | Was a relationship established prior to study commencement? | DH was involved in the recruiting of the nursing homes and in the initial discussions with the management of the participating nursing homes. |
| 7. Participant knowledge of the interviewer | What did the participants know about the researcher? | Participants knew about the researchers‘ affiliation. |
| 8. Interviewer characteristics | What characteristics were reported about the interviewer/facilitator? | None. |
| **Domain 2: Study design** | | |
| Theoretical framework | | |
| 9. Methodological orientation and Theory | What methodological orientation was stated to underpin the study? | Directed content analysis; interpretive evaluation. |
| Participant selection | | |
| 10. Sampling | How were participants selected? | N/A (secondary data analysis). |
| 11. Method of approach | How were participants approached? | Telephone and face-to-face. |
| 12. Sample size | How many participants were in the study? | 34 telephone interviews with 9 participants and 15 focus group interviews with 146 participants in total. |
| 13. Non-participation | How many people refused to participate or dropped out? Reasons? | N/A. |
| Setting | | |
| 14. Setting of data collection | Where was the data collected? | Workplace. |
| 15. Presence of non-participants | Was anyone else present besides the participants and researchers? | No. |
| 16. Description of sample | What are the important characteristics of the sample? | The mean age of the different participant groups (telephone interviewees, steering group members, moderators, nursing team members) ranged between 40 and 45.3 years, the mean working years between 10.2. and 20.8 and the mean time of employment in the nursing home between 6.2 and 11.8 years. 122 participants were female, 30 male, 3 not reported. 72 interviewees were geriatric nurses, 25 nurses, 27 nursing assistants and 27 others, 4 not reported. 100 of the participants were working full-time. |
| Data collection | | |
| 17. Interview guide | Were questions, prompts, guides provided by the authors? Was it pilot tested? | The overall interview topics were reported. The interview guide was pilot tested in a non-participating nursing home. |
| 18. Repeat interviews | Were repeat interviews carried out? If yes, how many? | No. |
| 19. Audio/visual recording | Did the research use audio or visual recording to collect the data? | Data were audio recorded using a digital recorder. |
| 20. Field notes | Were field notes made during and/or after the interview or focus group? | No. |
| 21. Duration | What was the duration of the interviews or focus group? | Telephone interviews: 15 minutes on average  Focus group interviews: 40 minutes on average. |
| 22. Data saturation | Was data saturation discussed? | N/A (secondary data analysis). |
| 23. Transcripts returned | Were transcripts returned to participants for comment and/or correction? | No. |
| **Domain 3: Analysis and findings** | | |
| Data analysis | | |
| 24. Number of data coders | How many data coders coded the data? | 1 (ST). |
| 25. Description of the coding tree | Did authors provide a description of the coding tree? | The coding tree is equivalent to the CFIR domains I, III and V, incl. (sub-) constructs, see Supplementary Table 2. |
| 26. Derivation of themes | Were themes identified in advance or derived from the data? | On the basis of the deductive coding, themes were interpretatively derived from the data. |
| 27. Software | What software, if applicable, was used to manage the data? | MAXQDA 2018. |
| 28. Participant checking | Did participants provide feedback on the findings? | No. |
| Reporting | | |
| 29. Quotations presented | Were participant quotations presented to illustrate the themes / findings? Was each quotation identified? | Participant quotations were presented. Each quotation was identified by referring to the nursing home and interview number. |
| 30. Data and findings consistent | Was there consistency between the data presented and the findings? | Yes. |
| 31. Clarity of major themes | Were major themes clearly presented in the findings? | Yes. |
| 32. Clarity of minor themes | Is there a description of diverse cases or discussion of minor themes? | Yes. |
