# Supplementary material for: Types of implementation of the dementia-specific case conference concept WELCOME-IdA in nursing homes: a qualitative process evaluation of the FallDem effectiveness trial
Source: Implement Sci Commun. 2021 Aug 18;2:90. doi: 10.1186/s43058-021-00191-0 (PMC8371834; doi:10.1186/s43058-021-00191-0)
Supplement: Supplementary file 2 — Additional file 2. [file 43058_2021_191_MOESM2_ESM.docx]

**Supplementary Table 2. Domains I, III & V of the Consolidated Framework for Implementation Research (CFIR).**^[[1]](#footnote-1)^

| **I. Intervention Characteristics** | | |
| --- | --- | --- |
| A | Intervention Source | Perception of key stakeholders about whether the intervention is externally or internally developed. |
| B | Evidence Strength & Quality | Stakeholders’ perceptions of the quality & validity of evidence supporting the belief that the intervention will have desired outcomes. |
| C | Relative Advantage | Stakeholders’ perception of the advantage of implementing the intervention versus an alternative solution. |
| D | Adaptability | The degree to which an intervention can be adapted, tailored, refined, or reinvented to meet local needs. |
| E | Trialability | The ability to test the intervention on a small scale in the organization, & to be able to reverse course (undo implementation) if warranted. |
| F | Complexity | Perceived difficulty of implementation, reflected by duration, scope, radicalness, disruptiveness, centrality, & intricacy & number of steps required to implement. |
| G | Design Quality & Packaging | Perceived excellence in how the intervention is bundled, presented, & assembled. |
| H | Cost | Costs of the intervention & costs associated with implementing the intervention including investment, supply, & opportunity costs. |
| **III. Inner Setting** | | |
| A | Structural Characteristics | The social architecture, age, maturity, & size of an organization. |
| B | Networks & Communications | The nature & quality of webs of social networks & the nature & quality of formal & informal communications within an organization. |
| C | Culture | Norms, values, & basic assumptions of a given organization. |
| D | Implementation Climate | The absorptive capacity for change, shared receptivity of involved individuals to an intervention, & the extent to which use of that intervention will be rewarded, supported, & expected within their organization. |
|  | *1 Tension for Change* | The degree to which stakeholders perceive the current situation as intolerable or needing change. |
|  | *2 Compatibility* | The degree of tangible fit between meaning & values attached to the intervention by involved individuals, how those align with individuals’ own norms, values, & perceived risks & needs, & how the intervention fits with existing workflows & systems. |
|  | *3 Relative Priority* | Individuals’ shared perception of the importance of the implementation within the organization. |
|  | *4 Organizational Incentives & Rewards* | Extrinsic incentives such as goal-sharing awards, performance reviews, promotions, & raises in salary, & less tangible incentives such as increased stature or respect. |
|  | *5 Goals & Feedback* | The degree to which goals are clearly communicated, acted upon, & fed back to staff, & alignment of that feedback with goals. |
|  | *6 Learning Climate* | A climate in which: a) leaders express their own fallibility & need for team members’ assistance & input; b) team members feel that they are essential, valued, & knowledgeable partners in the change process; c) individuals feel psychologically safe to try new methods; & d) there is sufficient time & space for reflective thinking & evaluation. |
| E | Readiness for Implementation | Tangible & immediate indicators of organizational commitment to its decision to implement an intervention. |
|  | *1 Leadership Engagement* | Commitment, involvement, & accountability of leaders & managers with the implementation. |
|  | *2 Available Resources* | The level of resources dedicated for implementation & on-going operations, including money, training, education, physical space, & time. |
|  | *3 Access to Knowledge & Information* | Ease of access to digestible information & knowledge about the intervention & how to incorporate it into work tasks. |
| **V. Process** | | |
| A | Planning | The degree to which a scheme or method of behavior & tasks for implementing an intervention are developed in advance, & the quality of those schemes or methods. |
| B | Engaging | Attracting & involving appropriate individuals in the implementation & use of the intervention through a combined strategy of social marketing, education, role modeling, training, & other similar activities. |
|  | *1 Opinion Leaders* | Individuals in an organization who have formal or informal influence on the attitudes & beliefs of their colleagues with respect to implementing the intervention. |
|  | *2 Formally Appointed Internal Implementation Leaders* | Individuals from within the organization who have been formally appointed with responsibility for implementing an intervention as coordinator, project manager, team leader, or other similar role. |
|  | *3 Champions* | “Individuals who dedicate themselves to supporting, marketing, & ‘driving through’ an [implementation]”^[[2]](#footnote-2)^, overcoming indifference or resistance that the intervention may provoke in an organization. |
|  | *4 External Change Agents* | Individuals who are affiliated with an outside entity who formally influence or facilitate intervention decisions in a desirable direction. |
|  | *5 Key Stakeholders* | e.g. providers & staff (proposed new sub-construct, will be included in CFIR V2) |
|  | *6 Innovation Participants* | e.g. patients (proposed new sub-construct, will be included in CFIR V2) |
| C | Executing | Carrying out or accomplishing the implementation according to plan. |
| D | Reflecting & Evaluating | Quantitative & qualitative feedback about the progress & quality of implementation accompanied with regular personal & team debriefing about progress & experience. |

1. Source: https://cfirguide.org/. [↑](#footnote-ref-1)
2. Greenhalgh, Trisha et al. 2004. ‘How to spread good ideas’, A systematic review of the literature on diffusion, dissemination & sustainability of innovations in health service delivery & organization. Report for NCCSDO: 1-424. [↑](#footnote-ref-2)
